# Using genetic variation in *Aedes aegypti* to identify candidate anti-dengue virus genes

**DOI:** 10.1186/s12879-019-4212-z

**Published:** 2019-07-04

**Authors:** Gerard Terradas, Elizabeth A. McGraw

**Affiliations:** 1 School of Biological Sciences, Monash University, Melbourne, Victoria 3800 Australia; 2 Center for Infectious Disease Dynamics, Department of Entomology, The Huck Institutes of the Life Sciences, W251 Millennium Science Complex, The Pennsylvania State University, University Park, PA 16802 USA

**Keywords:** Genetic variation, Transcriptomics, *Aedes*, Dengue virus, Innate immunity

## Abstract

**Background:**

Transcriptomic profiling has generated extensive lists of genes that respond to viral infection in mosquitoes. These gene lists contain two types of genes; (1) those that are responsible for the insect’s natural antiviral defense mechanisms, including some known innate immunity genes, and (2) genes whose change in expression may occur simply as a result of infection. As genetic modification tools for mosquitoes continue to improve, the opportunities to make refractory insects via allelic replacement or delivery of small RNAs that alter gene expression are expanding. Therefore, the ability to identify which genes in transcriptional profiles may have immune function has increasing value. Arboviruses encounter a range of mosquito tissues and physiologies as they traverse from the midgut to the salivary glands. While the midgut is well-studied as the primary tissue barrier, antiviral genes expressed in the subsequent tissues of the carcass offer additional candidates for second stage intervention in the mosquito body.

**Methods:**

Mosquito lines collected recently from field populations exhibit natural genetic variation for dengue virus susceptibility. We sought to use a modified full-sib breeding design to identify mosquito families that varied in their dengue viral load in their bodies post infection.

**Results:**

By delivering virus intrathoracically, we bypassed the midgut and focused on whole body responses in order to evaluate carcass-associated refractoriness. We tested 25 candidate genes selected for their appearance in multiple published transcriptional profiles and were able to identify 12 whose expression varied with susceptibility in the genetic families.

**Conclusions:**

This method, using natural genetic variation, offers a simple means to screen and reduce candidate gene lists prior to carrying out more labor-intensive functional studies. The extracted RNA from the females across the families represents a storable resource that can be used to screen subsequent candidate genes in the future. The aspect of vector competence being assessed could be varied by focusing on different tissues or time points post infection.

**Electronic supplementary material:**

The online version of this article (10.1186/s12879-019-4212-z) contains supplementary material, which is available to authorized users.

## Background

The *Aedes aegypti* mosquito is the primary vector of dengue virus (DENV) to humans with roughly 40% of the world’s population at risk of infection [1]. The mosquito has been highly successful in colonizing the tropics and subtropics around the globe, recently spreading out of Africa, assisted by climate change and urbanization [2]. Despite this rapid expansion, there is evidence of local variation in mosquito susceptibility to DENV [3–6] and diversity of the possible mechanisms conferring refractoriness [7–10].

As DENV traverses the body of the mosquito, the capacity for viruses like DENV to interact with mosquito physiology is vast given the range of tissues and cell types encountered during the process of infection [11–13]. Viruses must first infect the mosquito midgut upon the consumption of a viral laden blood meal and then exit into the carcass. In the body, viruses infect a range of tissues including hemolymph, fat body, nerve and muscle [14, 15]. While the model for dengue viral progression is thought to be stepwise; midgut to organs in the carcass to the salivary glands, there is also evidence that virus may also spread to the rest of the body using the trachea [15]. The genetics of the midgut and salivary gland response have been particularly well studied, as they are thought to serve as particular barriers to virus progression [16, 17]. The carcass however contains the immune organs [18–20] and the bulk of the mosquito’s tissue mass where arboviruses are known to replicate [16]. Genes acting in the carcass tissues therefore offer potential opportunities to interfere with viral infection.

Several studies have profiled the transcriptional response of *Ae. aegypti* to DENV at different time points post infection and in a range of insect tissues [12, 13, 21]. These approaches have been instrumental in characterizing the nature of the insect’s humoral immune response [22]. In these studies, there can be thousands of genes exhibiting transcriptional change. It is difficult to disentangle whether changes are related to the host antiviral response, the physiological response of the vector to infection or direct modulation of host pathways by the virus. Additionally, while many insect innate immunity pathways have been mapped including both humoral [23–27] and cellular components [28, 29], it is also clear that large numbers of genes outside of these core pathways function in immunity in unknown ways.

Emerging insect genetic tools, including CRISPR-Cas9 [30–32] and microRNA targeting of gene expression [33] offer means for manipulating the protein coding sequence of key genes in the mosquito as well as altering their expression, respectively. These methods offer not only powerful ways to test individual gene function, but also potential applications in vector borne disease control [34]. Regardless of their ongoing development, these approaches are still labor intensive, as screening the thousands of genes responding to viral infection in vectors is impractical. Techniques for first decreasing the number of candidate genes in the workflow are therefore timely. Examination of candidate gene expression in diverse contexts (tissues, timepoints, viruses) as well as more manipulative scenarios using RNAi or siRNA in cell culture or mosquitoes, respectively offer two means.

Here we selected a set of mosquito candidate genes that were common responders to viral infection across a range of transcriptional profile studies. We then tested for associations between their expression and mosquitoes exhibiting genetic variation for DENV load in their body. While the initial quantitative genetic breeding design utilized for differentiating mosquito families was labor intensive, the RNA collected from individuals is a storable resource that can be revisited in the future to test the behavior of additional gene candidates.

## Methods

### Mosquito collection and rearing

Mosquitoes were collected by the Eliminate Dengue team associated with James Cook University from private properties with permission from the residents within and outside the Eliminate Dengue release zone in Greater Cairns, QLD, Australia. *Ae. aegypti* were identified by morphology and later checked by *Ae. aegypti-*specific qPCR primer detection [35]. These wildtype mosquitoes were confirmed not to harbor *Wolbachia* infection by PCR [35]. Mosquitoes were hatched and reared at a density of ~ 150 larvae in 30 × 40 × 8 cm trays containing 3 L of RO water in controlled conditions of temperature (26 ± 2 °C), humidity (~ 70%) and photoperiod (12:12, light:dark). Larvae were fed fish food (Tetramin, Melle, Germany). Males and females were sexed after pupation and transferred separately to 30 × 30 × 30 cm cages to allow eclosion at a density of ~ 450 individuals/cage. Adult mosquitoes were kept on a 10% sucrose water diet. Six to eight day old adult females (P1) were group fed on human volunteers. A modified full sib breeding design was performed as depicted in a previous paper [36] and yielded 25 independent wildtype mosquito families. In brief, parental single pair crosses (male with a virgin female) were set up and those that exhibited sufficient egg production were selected for F_1_ intercrossing and progressed to F_2_. DENV-2 was then injected intrathoracically into 6–7 day old F_2_ mosquitoes and either tissues (head, ovaries, midgut and rest of the body) or whole mosquitoes were collected at 7 days post infection to evaluate both DENV-2 loads and candidate gene expression.

### Constraints of working with a family-based breeding design

Experiments involving any type of family-based breeding design must contain large numbers of families and individuals per family to obtain sufficient statistical power to detect differences. Mosquito families are easily disqualified from such designs due to poor oviposition or hatch ranges. Despite that, we obtained 25 families showing a wide range of DENV loads, from which only the extremes were progressed for further study. When evaluating traits like viral loads and gene expression, which can vary immensely between time points, synchronizing and controlling the mosquitoes’ age is essential. Families were infected over a period of 2 days. Intrathoracic injection also allowed us to deliver the same amount of virus to all individuals in a controlled manner. DENV load at 7 dpi did not vary with respect to whether the mosquitoes had been injected on the first or second day of infections. Only a single survey point (7dpi) was possible given the size of the experiment and the number of samples needed for statistical power. This time point is routinely used for vector competence experiments because infections have disseminated [15] and it is close to the average extrinsic incubation period (EIP), or time to appearance of virus in saliva, of wildtype mosquito populations [36, 37]. As intrathoracic viral delivery speeds the process of infection, mosquitoes would likely be in a slightly later stage of DENV infection.

### Virus intrathoracic injections

Intrathoracic injections were used to focus on anti-DENV processes that are body wide, rather than midgut focused and to avoid local blood meal associated changes in gene expression. A dengue virus serotype 2 strain (DENV-2, ET300) isolated from human serum collected from patients from East Timor in 2000 was used for intrathoracic injections. Virus was propagated and collected in cell culture as described previously [38]. Briefly, virus was inoculated into C6/36 cells grown in RPMI 1640 (Invitrogen) supplemented with 1 x Glutamax (Invitrogen) and 2% FBS and buffered with 25 mM HEPES (Sigma-Aldrich). Seven days post inoculation, virus was collected from the supernatant by centrifugation at 3200 g for 15 min at 4 °C. Viral stocks were stored at − 80 °C until further use and titrated using plaque assays. *Ae. aegypti* females were anesthetized with CO_2_ and 59 nL of DENV (~ 70 DENV-2 pfu) were injected intrathoracically using a pulled glass capillary with a manual microinjector (Nanoject II, Drummond Sci., Broomall, PA, USA). The concentration was selected from pilot studies to be sure all mosquitoes had high viral loads. Virus stock was diluted to the desired concentration using culture RPMI media. After injection, mosquitoes were maintained under identical initial controlled conditions as per above.

### RNA extractions

In addition to 327 dissected females, 171 whole mosquitoes were collected as individuals (not pools) at 7 days post injection and extracted using TRIzol (Invitrogen, Carlsbad, CA, USA). Mosquito families included from 5 to 15 individuals, that were each dissected for head, midgut, ovary and salivary glands. For families with greater than 15 individuals, the remainder were collected as whole mosquitoes. All were samples homogenized using a TissueLyser II (Qiagen, Hilden, Germany) and stored at − 80 °C until further use. RNA was extracted following the manufacturer’s instructions. RNA yield was quantified using a Nanodrop™ Lite Spectophotometer (ThermoFisher Scientific, Waltham, MA, USA). RNA samples were stored at − 80 °C. Heads were used initially to survey for viral infection, but whole bodies were preferred for gene expression analyses as they are likely to capture a broader suite of genes involved with the infection response across the diverse tissues.

### DENV analysis

RNA samples were diluted to a concentration of 10 ng/μl prior to DENV qPCR analysis. One-step quantitative RT-PCR (qRT-PCR) to detect DENV loads was performed using TaqMan® Fast Virus 1-step Master Mix (Roche Applied Science, Switzerland) in a total volume of 10 μl and following manufacturer’s instructions on a LightCycler480 (Roche Applied Science, Switzerland). DENV qRT-PCR reactions were performed as described previously [39]. The number of viral copies present in each sample was evaluated using known standards [40]. The used standards ranged from 10^8^ to 10 DENV fragment copies. The limit of detection was set at 100 DENV copies as virus. Concentration of DENV in each sample adjusted to DENV copies/μg of total RNA using the standard curve. Standards and samples were run in duplicate.

### Candidate selection

We carried out a literature search in Pubmed [41] using the terms ‘dengue virus’ AND ‘Aedes’ AND ‘expression’ OR ‘transcriptional profile’ in March of 2017 to identify all transcriptomic studies examining the mosquito genetic response to dengue virus infection [9, 12, 13, 21, 42]. The studies commonly involved surveys across a range of time points post infection and in diverse tissues. The candidates we selected for testing satisfied at least one of the following criteria: the gene demonstrated significant change in expression in more than one transcriptomic profile; the gene exhibited significant expression change in a single transcriptome but has been previously unexplored in the literature for a role in DENV control; the gene showed high differential expression at 7dpi, but not at any other surveyed time point. The original source(s) for each candidate gene and direction of expression modulation is shown in Table 1.

**Table 1:** Candidate genes tested and their relevance in DENV control

Accession number	Gene name	Transcriptomic study	Function	Tissue	Direction	Differentially expressed	Interfamily variation
AAEL001022	*smp-30/regucalcin*	[12, 21]	Ca^2+^ binding domain	Whole	Down	Yes	-
AAEL001156	*CG5280*	[12, 13]	-	Whole	Up	No	Yes
AAEL001392	*defA-assoc*	[11–13, 20]	Immunity	Whole, Carcass	Down/Up	Yes	-
AAEL002413	*sphingomyelin*	[12]	Cellular membrane	Whole	Down	Yes	-
AAEL002585	*CLIPA11*	[12, 42]	Serine protease	Whole, MG	Down	No	Yes
AAEL003619	*-*	[12, 21]	Na/Cl transporter	Whole	Down	Yes	-
AAEL003787	*Nopo*	[12]	Zinc finger	Whole	Up	Yes	-
AAEL004361	*alpha-glucosidase*	[12]	Glycolysis	Whole	Down	Yes	-
AAEL004861	*degringolade*	[12, 13]	Peroxisomal integral protein	Whole, Carcass	Up	No	Yes
AAEL005064	*CLIPB5*	[12, 21]	Serine protease	Whole	Down	No	Yes
AAEL005527	*Nbr/mut-7*	[12, 13, 21]	miRNA maturation	Whole, SG	Down	No	Yes
AAEL006995	*CG9657*	[12, 21]	-	Whole	Down	No	No
AAEL007495	*phosphoglycerate mutase*	[12, 13, 21]	Glycolysis	Whole, Carcass, MG	Down	No	No
AAEL007845	*Rab5*	-	Receptor	-	-	Yes	-
AAEL008013	*Obp83b*	[12, 21]	Odorant	Whole	Down	No	Yes
AAEL008108	*GB76c*	[12, 13, 21]	Transmembrane signalling	Whole, Carcass, SG	Down	Yes	-
AAEL009317	*Rab11*	[12]	GTPase, cellular trafficking	Whole	Up	No	No
AAEL009602	*Gdap1*	[11, 12, 21]	Mitochondrial membrane	Whole, Midgut	Down	No	No
AAEL009770	*SUMOE2*	[12]	Sumoylation	Whole	Up	Yes	-
AAEL011375	*trypsin*	[12, 13, 21]	Serine protease	Whole, Carcass	Down	Yes	-
AAEL011566	*-*	[11, 12, 21]	Adhesion	Whole, Carcass, MG	Down	Yes	-
AAEL011817	*rent1*	[12]	mRNA decay	Whole	Down	No	Yes
AAEL012089	*xport-A*	[11, 12, 21]	Phototransduction	Whole, Carcass	Down	No	No
AAEL013712	*Trypsin 5G1 precursor*	[11, 12, 21]	Serine protease	Whole, Carcass	Up	No	No
AAEL014108	aquaporin	[12, 13, 21]	H_2_O Transporter	Whole, Carcass	Down	Yes	-

Accession numbers, gene names, function and patterns of expression across families with high and low DENV loads for the tested genes. All had been previously reported in transcriptomic studies associated with differences in expression in at least two conditions (tissues/timepoints). Tissues of previous reported expression differences are shown. In “Tissue”, MG and SG correspond to midgut and salivary glands, respectively. Whether the genes were up or downregulated in those studies can be seen in “Direction”, and our reported expression patterns are shown in the column “Differentially expressed” as well as “Interfamily Variation”

### Candidate gene expression

SuperScript® III Reverse Transcriptase kit (Invitrogen, Carlsbad, CA, USA) was used to convert RNA to cDNA in all carcass samples. The reaction contained 12.5 μl of RNA undiluted template, 1 μl of random primers (RP, 125 ng/μl), 1 μl of deoxynucleotides (dNTPs, 2.5 mM), dithiothreitol (DTT), 5X buffer and enzyme as per kit instructions, with a total volume of 20 μl. cDNA synthesis was performed in a C1000™Thermal Cycler (Bio-Rad, Hercules, CA, USA) on the following temperature profile: 5′ at 65 °C followed by 10′ at 25 °C, 50′ at 50 °C, 10′ at 75 °C and kept at 4 °C. Gene expression levels were detected with SYBR® Green I Master (Roche Applied Science, Switzerland) using 1.5 μl of a 1:5 dilution from the previously synthesized cDNA on a LightCycler480 (Roche Applied Science, Switzerland). Corresponding Ct values were normalized to the housekeeping *Ae. aegypti RpS17* gene [43] and expression ratios obtained using the ∆∆Ct method [44]. Primer sequences for candidate genes can be found in Additional file 1: Table S1.

### Statistical analysis

DENV loads and gene expression data were analyzed using a generalized mixed model with a random factor ‘Family’ nested with ‘disseminated’ DENV load, with the latter also set as a fixed factor. Statistics were performed using IBM SPSS Statistics (v23) and graphs created using Prism 7 (GraphPad Software Inc., San Diego, CA, USA).

## Results

### DENV load classification

**Fig. 1:**
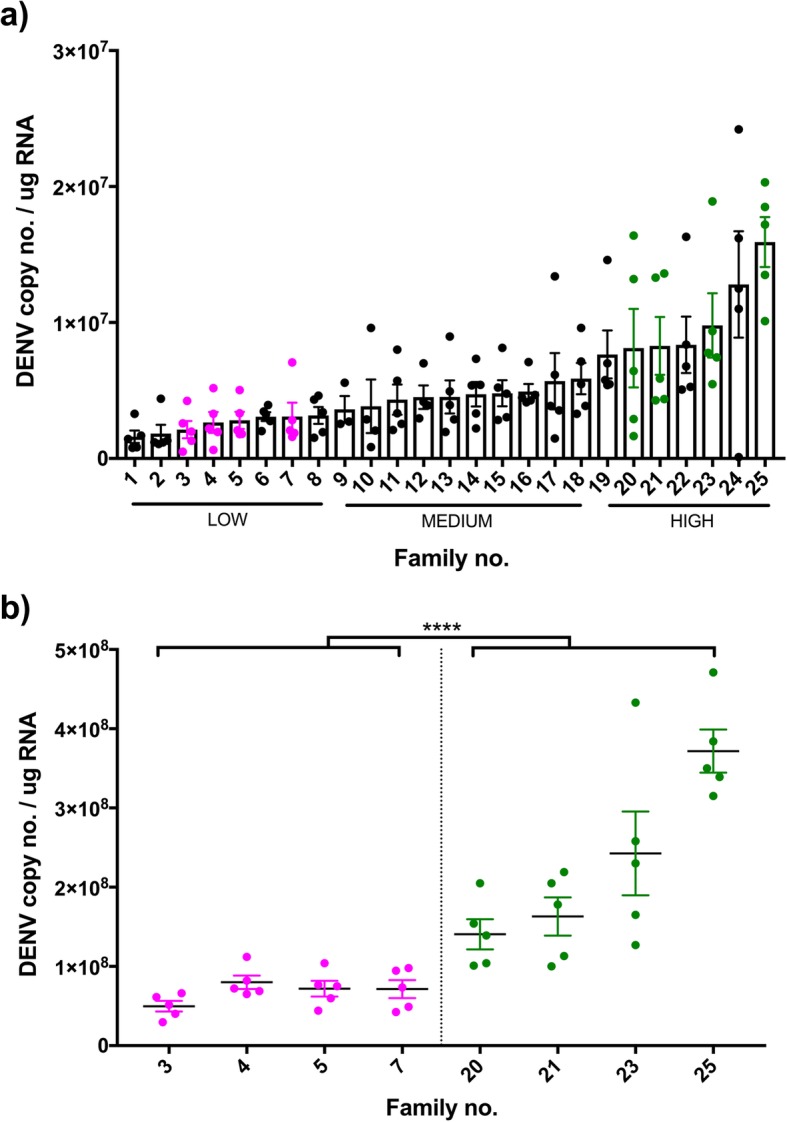
Disseminated DENV loads. Wildtype DENV-infected families were classified based on head DENV loads; families that were progressed to gene expression analyses are highlighted in pink (Low DENV load) or green (High DENV load) (**a**). DENV phenotype was later confirmed with whole body load (**b**). Each dot in the graph depicts a single mosquito. Bars depict family DENV mean and SEM

We performed a modified full sib breeding design on an Australian population of *Aedes aegypti* to determine the nature of the genetic variation for DENV susceptibility. We assessed the load of DENV serotype 2 in the head tissue of females 7 days post intrathoracic injection of virus (Fig. 1a) for 25 families of mosquitoes. We then selected a range of families representing the extremes in DENV load (4 each) and confirmed that these differences were also seen in whole body measures of DENV load in sisters from the same families (Fig. 1b), which were later used for gene expression analyses. We used a nested generalized linear model (GLiM) to assess differences in total DENV loads between our extreme families, where ‘Family number’ was nested within ‘DENV load’ (High or Low, in heads). We observed a significant effect of ‘DENV load’ (Wald = 104.08, df = 1, *p* < 0.0001), supporting our designation of families as High or Low. We also observed a ‘Family within DENV load’ effect (Wald = 81.97, df = 6, p < 0.0001) that relates to the presence of interfamily variation for the trait, especially in the High DENV group.

After demonstrating that DENV load varied between groups, we selected a subset of 4 families each representing the phenotypic extremes of DENV load to test for associations with expression of candidate antiviral genes in whole bodies. Genes tested (Table 1) stem from previous transcriptomic studies, but have yet to be confirmed by further functional studies. A range of genes (roughly half of those tested) representing diverse functional classes did not exhibit patterns of expression across families that would explain differences in DENV load. Other genes, while exhibiting mean expression patterns consistent with DENV control, also exhibited a large amount of variation between families within a phenotypic class and hence could not be interpreted (data available on Figshare). These genes may be highly influenced by environmental or epistatic effects. Neither of these classes of genes would represent good candidates for subsequent genetic modification. Below we present the data from genes exhibiting uniformity of response across families within the phenotypic extremes and that differed with respect to DENV load.

### Immune genes and signaling

Host immune responses are one of the main contributors to mosquito pathogen control [22]. Successful bacteria and viruses are able to promote transcription of proteins that suppress key host immune responses, in order for the pathogen to replicate and proliferate freely. Such proteins can be classified in three main ways; molecules that the virus uses as cofactors to replicate (1), molecules involved in cell signaling (2) that in turn activate immune pathways to promote transcription of immune effectors (3).

**Fig. 2:**
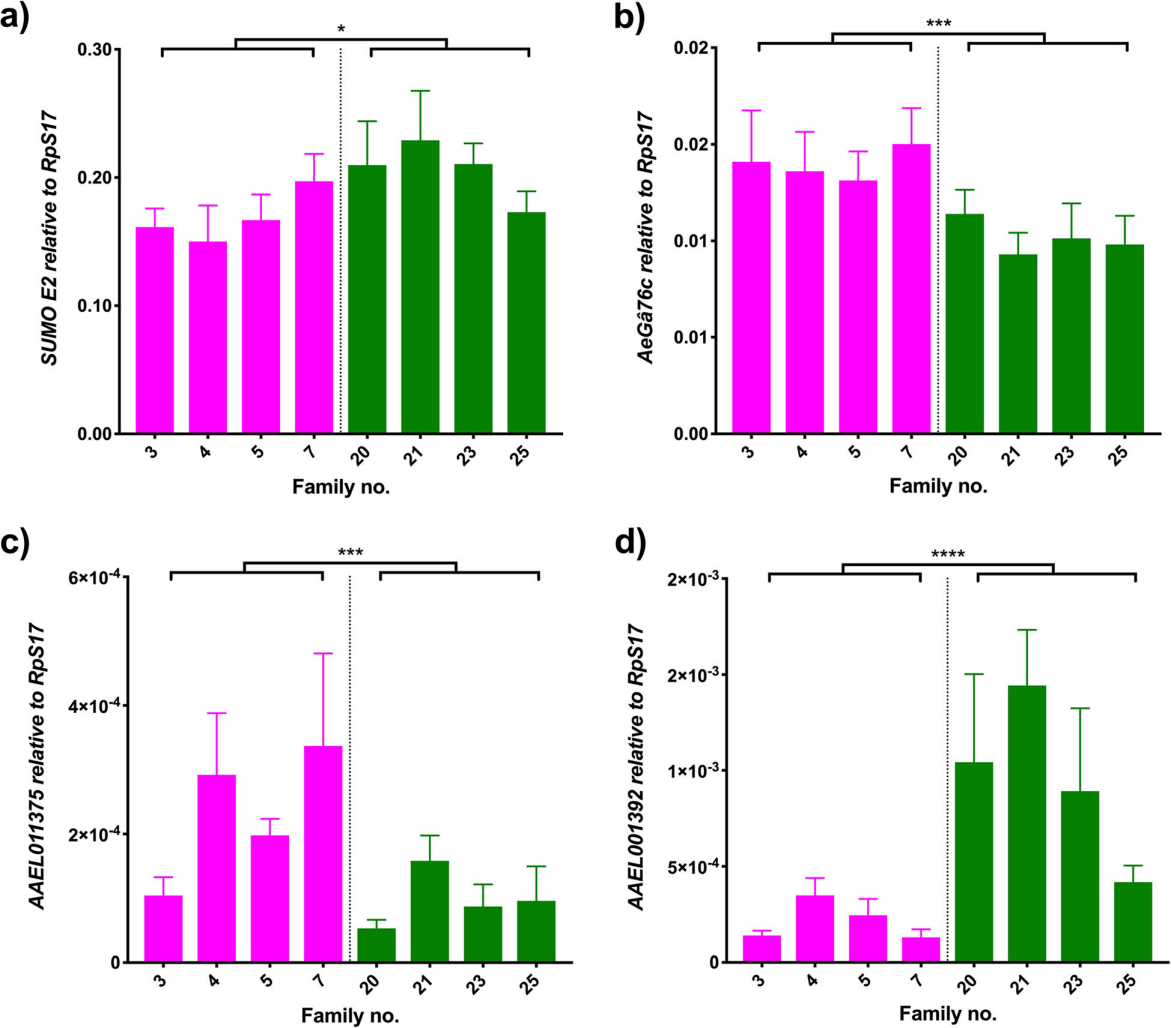
Immune gene and signalling. Graphs show the expression of (**a**) *SUMOE2*, (**b**) *AeG*α*76C,* (**c**) *AAEL011375* and (**d**) *AAEL001392* relative to *RpS17* in DENV-infected individuals. Pink bars represent refractory families; green bars represent susceptible families. Bars depict family mean and SEM (n = 5). * 0.05 < p < 0.01, ***0.001 < 0.0001, **** p < 0.0001. The average fold change of refractory/susceptible families is 0.82 (**a**), 1.37(**b**), 2.36 (**c**), 0.22 (**d**)

From the first group, SUMOE2 is a protein with a range of effects on the host, whose high levels have also been linked to increased DENV loads in human cells, as the virus uses sumoylation to tag its NS5 and regulate replication via the suppression of antiviral responses [45]. In our study, the expression of the gene *AeSUMOE2* (*AAEL009770*) had a significant effect of DENV load (Fig. 2a; Wald = 5.34, df = 1, *p* = 0.021) and no significant difference was seen between families in each DENV group (Wald = 5.68, df = 6, *p* = 0.46), suggesting that *AeSUMOE2* plays a role in DENV control. This is in keeping with the observations from previous transcriptomic studies, where a slight increase in *AeSUMOE2* expression was seen in hosts infected with DENV.

We also evaluated the contributions of two proteins that act as signaling molecules, AeGβ76C (*AAEL008108*) and a serine protease (*AAEL011375*), and one effector, *AAEL001392*. Little is known about *AeGβ76C* expression, involved in rhodopsin and signal transduction, but we detected a significant effect based on DENV load (Fig. 2b; Wald = 11.4, df = 1, *p* = 0.001) but not differences within families of each group (Wald = 1.7, df = 6, *p* = 0.945). Similar effects are seen for *AAEL011375*, where DENV load group effect was significant (Fig. 2c; Wald = 9.69, df = 1, *p* = 0.002) but no effect of family within DENV load was detected (Wald = 10.17, df = 6, *p* = 0.118). Both expression levels correlate with the downregulation seen in previous transcriptomic studies. The expression of AAEL001392, however, does not match the modulation observed in transcriptomic profiles. We observed a significant DENV load effect (Fig. 2d; Wald = 21.42, df = 1, *p* < 0.0001) and no effect of family within DENV load (Wald = 11.22, df = 6, *p* = 0.082), but the direction of the main effect is the opposite of that observed in transcriptomic studies, which suggest that its expression is down regulated by the virus, despite other functional studies showing upregulation of the immune effector in response to the viral infection [24].

### Apoptosis genes

**Fig. 3:**
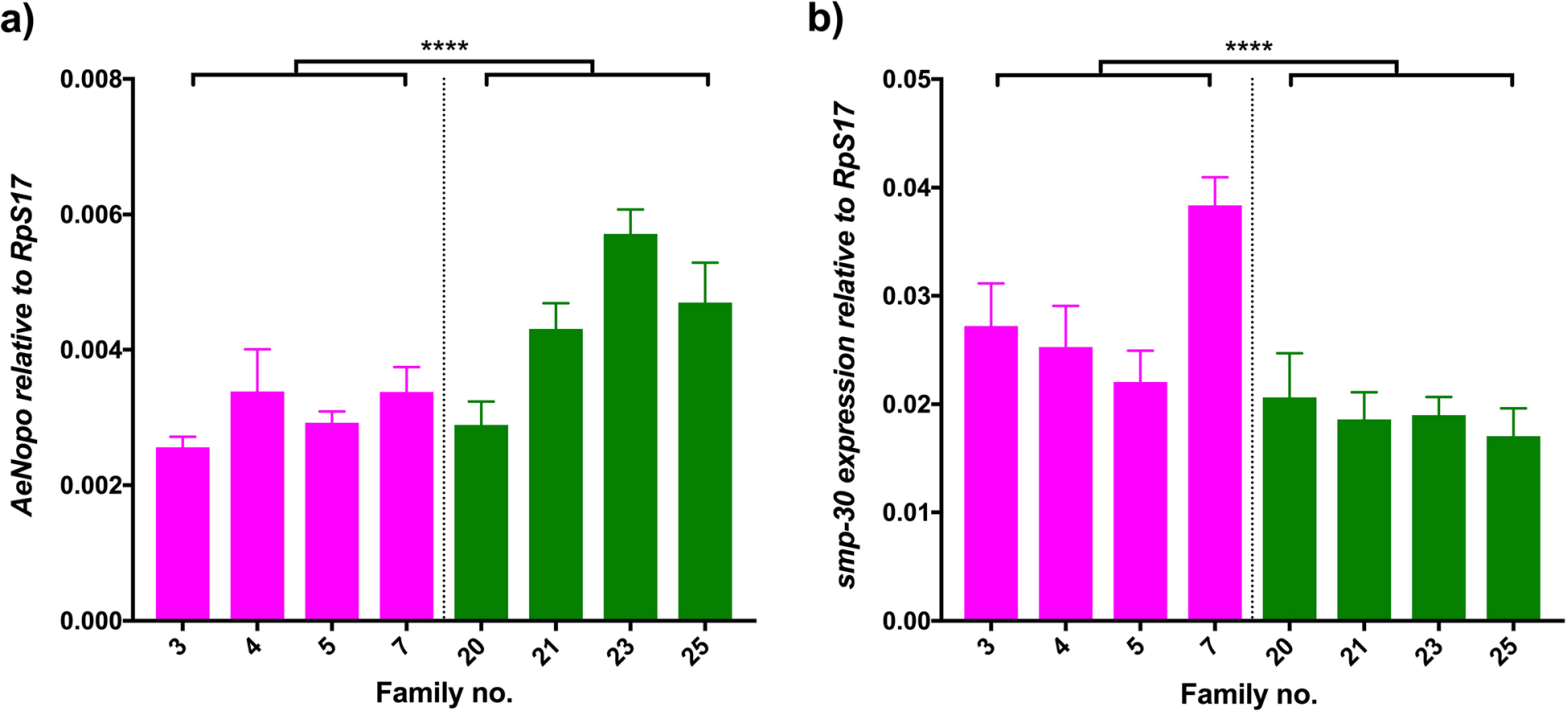
Apoptosis. Graphs show the expression of (**a**) *AeNopo* and (**b**) *smp-30* relative to *RpS17* in DENV-infected individuals. Pink bars represent refractory families; green bars represent susceptible families. Bars depict family mean and SEM (*n* = 5). **** *p* < 0.0001. The average fold change of refractory/susceptible families is 0.69 (**a**), 1.49 (**b**)

Classic signaling immune pathways are not the only responses that the host mount against an incoming pathogen. Different immune pathways usually act synergistically with apoptotic responses to determine infection outcomes [21, 46]. There have been previous studies that focus on the role of apoptosis-related genes and their relevance to viral control [29, 47, 48], where increased cellular death promotes replication. We evaluated two genes involved in the regulation of apoptosis, *AeNopo* (*AAEL003787*) and the *senescence marker protein 30* (*smp-30/regucalcin*, *AAEL001022*). *AeNopo* is a zinc finger domain that directly regulates caspase activity and thus its upregulation promotes cell death via activation of pro-apoptotic genes [49]. We observed a significant upregulation of *AeNopo* in highly infected families (Fig. 3a; Wald = 27.34, df = 1, *p* < 0.0001). The variation of the expression in families of the same DENV group was also significant (Wald = 34.76, df = 6, p < 0.0001), suggesting that levels can vary greatly between genotypes. *Smp-30* regulates cellular Ca^2+^ homeostasis and has a role in cellular protection against oxidative stress, which has been linked to DENV infection status [50]. We observed a significant downregulation of *smp-30* between DENV groups (Fig. 3b; Wald = 22.89, df = 1, *p* < 0.0001). The variation of the expression in families of the same DENV group was also significant (Wald = 20.37, df = 6, *p* < 0.002). Both *AeNopo* and *smp-30* data bode well with the transcriptomic patterns seen in previous studies.

### Metabolism genes

**Fig. 4:**
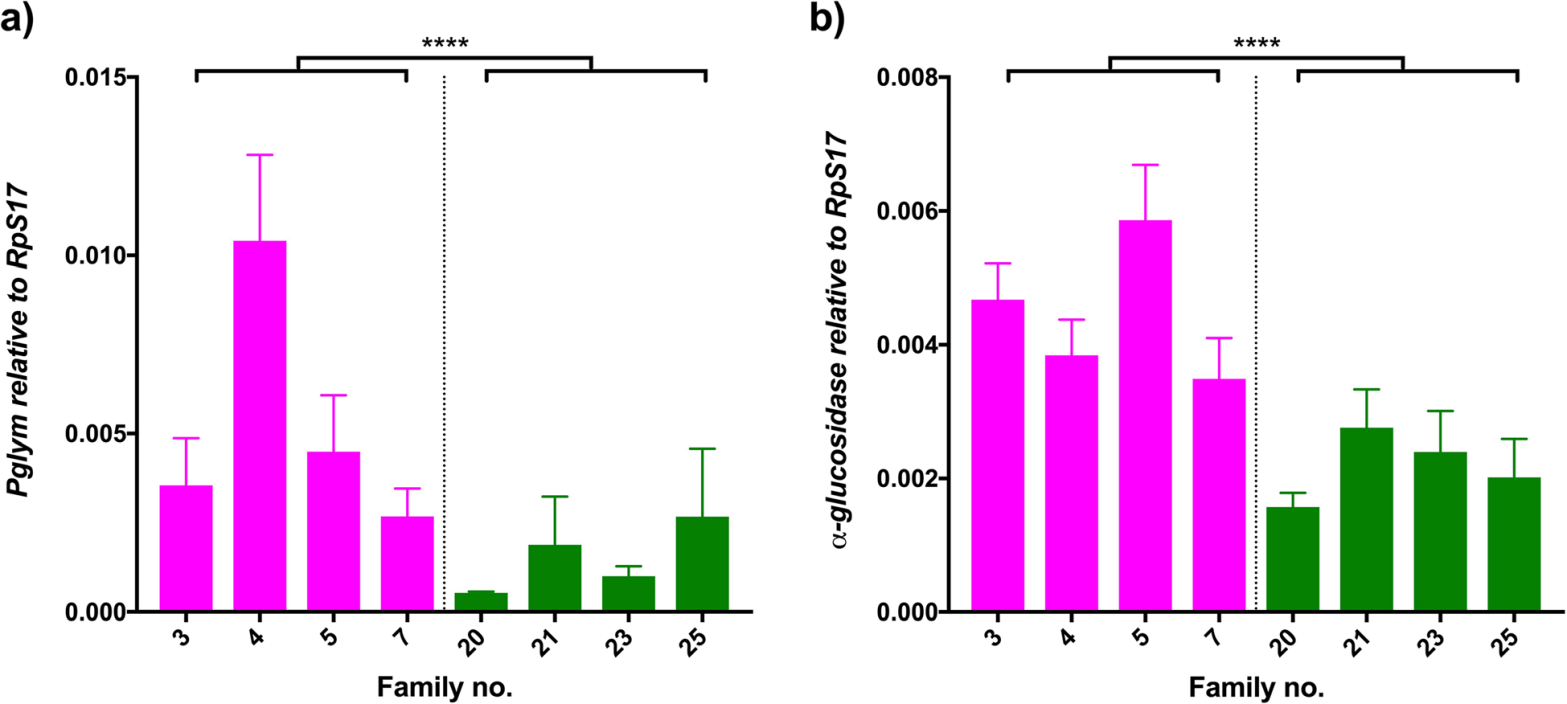
Metabolism. Graphs show the expression of (**a**) *Pglym* and (**b**) α*-glucosidase* relative to *RpS17* in DENV-infected individuals. Pink bars represent refractory families; green bars represent susceptible families. Bars depict family mean and SEM (n = 5). **** p < 0.0001. The average fold change of refractory/susceptible families is 3.47 (**a**), 2.04 (**b**)

Another effect that DENV has on host cells is the modulation of lipid metabolism and its cellular homeostasis. This may be caused by the virus relying on host structures to assemble its own replication machinery, a required modulation of membranes to facilitate viral infection or a mechanism to promote intracellular virion trafficking [51–54]. We analyzed two different molecules involved in metabolism of lipids and sugars that were identified as down regulated in response to a DENV-infected blood meal. In concordance with transcriptomic studies, phosphoglycerate mutase (*Pglym*, *AAEL007495*) was observed to be down regulated in highly infected families (Fig. 4a, Wald = 17.47, df = 1, *p* < 0.0001) and so was α-glucosidase *(*α*-gluc*, *AAEL004361*) (Fig. 4b, Wald = 38.31, df = 1, p < 0.0001). Both genes’ expression was also significant when analyzing the variation between families of the same group (*Pglym*: Wald = 24.27, df = 6, p < 0.0001; α*-gluc*: Wald = 15.13, df = 6, *p* < 0.019).

### Transporter and adhesion genes

**Fig. 5:**
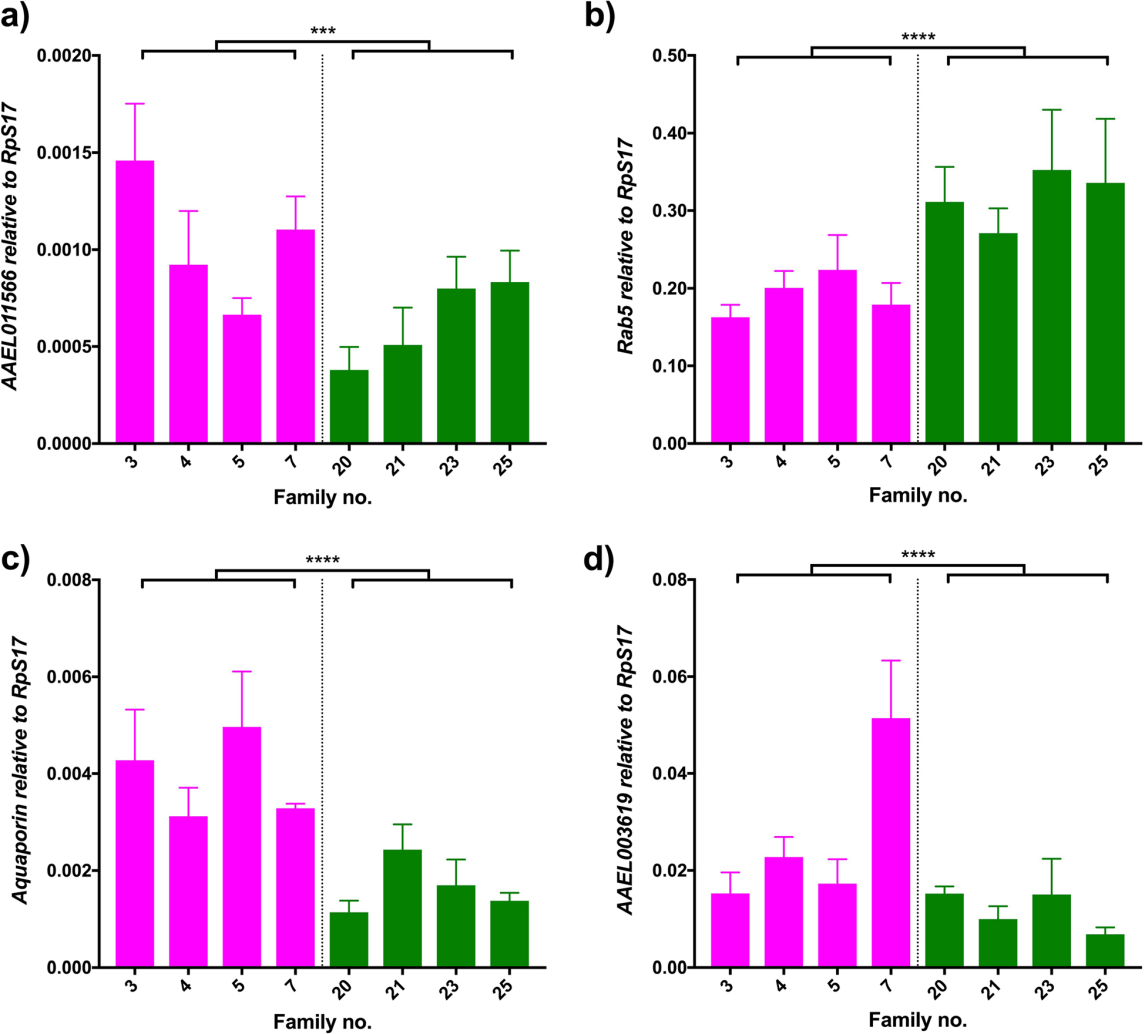
Adhesion and transport. Graphs show the expression of (**a**) *AAEL011566*, (**b**) *Rab5,* (**c**) *aquaporin* and (**d**) *AAEL003619* relative to *RpS17* in DENV-infected individuals. Pink bars represent refractory families; green bars represent susceptible families. Bars depict family mean and SEM (n = 5). *** 0.001 < p < 0.0001, **** p < 0.0001. The average fold change of refractory/susceptible families is 1.64 (**a**), 2.35 (**b**), 0.60 (**c**), 1.10 (**d**)

As mentioned previously, an essential component of the viral success is the attachment of the virion to the cell. After that, membrane fusion can occur and virus can start replicating inside the cytoplasm. We evaluated differences in expression for a range of intracellular transporters and molecules involved in cellular adhesion. From the latter, an uncharacterized adhesion molecule (*AAEL011566*) was highly down regulated in both transcriptomic studies on DENV infection and in the effect of bloodmeals in mosquitoes [11]. Our data supports its effects on DENV load, as its expression is down regulated in the high DENV load families (Fig. 5a; Wald = 10.95, df = 1, *p* < 0.0001). Variability among grouped families is also present, as expression differences within families from the same DENV load group are significant (Wald = 15.9, df = 6, *p* = 0.014). Due to its relevance to viral success, a broad range of molecules involved in adhesion and endocytosis has been characterized in functional studies. Despite no modulation was seen in transcriptomic studies for *Rab5* (*AAEL007845*), an endocytic molecule, it has been previously labelled as a required component for cellular entry of arboviruses [55–57]. We investigated whether differences in expression between low and highly infected families were present at a late infection timepoint. *Rab5* expression was significantly up regulated in families belonging to the high DENV load group (Fig. 5b; Wald = 16.34, df = 1, *p* < 0.0001) and no differences were found among families from the same DENV load group (Wald = 3, df = 6, *p* = 0.808).

We also analyzed two cellular transporters, *aquaporin* (*AAEL014108*) and a putative Na/Cl-dependent amino acid transporter (*AAEL003619*). Members of the aquaporin family are transmembrane molecules that transport water and other small solutes in and out of the cell, that may assist with seasonal adaptation and bloodmeal-induced diuresis [58, 59]. Aquaporin was one of the main candidates that arose from different transcriptomic studies [12, 21], showing expression downregulation at all sampled timepoints after arboviral challenge. Similarly, we observed a significant difference in expression for the main effect of DENV load (Fig. 5c; Wald = 29.83, df = 1, *p* < 0.0001) but no effect of family within DENV group was present (Wald = 9.46, df = 6, *p* = 0.149).

Downregulation of expression of *AAEL003619*, an amino acid transporter, may be due to the intracellular amino acid pool being used by the virus to replicate. The effect was significant for DENV load (Fig. 5d; Wald = 16.69, df = 1, p < 0.0001) and so was variation within each group, shown by the significance of the effect of family within DENV load (Wald = 33.54, df = 6, p < 0.0001).

## Discussion

Transcriptomic studies and other novel approaches that reveal differentially transcribed genes produce lists of candidates that can number in the thousands. When studying the response of a vector to viral infection, some of the gene candidates will be directly involved in viral control, whereas others may simply exhibit change in expression due to the cellular state of the vector. We know that many of the genes changing in response to infection actually do so in response to blood feeding [60, 61] and it is likely that other genes are responding to cellular damage caused by the virus [62]. Here we demonstrate the presence of genetic variation among mosquito families for body cavity DENV load and use this variation to screen candidate virus responder genes for those with possible anti/proviral activity.

We used this natural genetic variation in a mosquito population to specifically test whether candidate genes from previous transcriptomic studies may underpin differences in viral control in the vector. We intentionally focused on genes that had not been explored experimentally beyond transcriptional profiling. While we report genes that had distinct RNA expression profiles between families differing in DENV loads, genes that proved unimportant in our study could still play a role in viral control post-transcriptionally. By mapping the expression of candidate genes across families with extremes in vector competence, we were able to identify 12 out of 25 genes whose expression correlates with viral control in our families. These genes corresponded to three clusters of functional classes involved in viral control: immunity, adhesion and intracellular transport and metabolism, reaffirming the importance of these three components to overall infection outcomes.

### Immunity

DENV actively modulates host cellular processes to establish infection and propagate [63, 64]. The host in turn responds with a range of known antiviral effects, mostly via activation of the innate immune system [24, 26, 65]. Previously, Toll activity has been shown to be required for DENV control in mosquitoes [24] and increased early antiviral protection can be observed in individuals with higher basal activation levels of the pathway [9]. Interestingly, transcriptomic studies demonstrate that a protein likely to be associated to one of the pathway’s effector genes, *defensin,* is routinely down regulated in response to DENV infection [12, 21]. Our results, however, show an upregulation of the gene’s expression in families with high DENV loads. This discrepancy may be due to differences in mosquito genotypes, sample time post infection or other factors that vary across studies. Expression of immune effectors varies highly depending on the time post infection [46, 66].

The genes *SUMOE2* (Fig. 2a), *AeNopo* and *smp-30* (Fig. 3) are thought to be involved in modification processes and apoptotic responses. The former plays a role in sumoylation, a process that stabilizes non-structural DENV proteins for proper replication [45] as well as modification of host proteins [67, 68]. The latter two genes are involved in apoptosis, a cellular death process that promotes DENV replication [47, 69]. *AeNopo* (Fig.3a) has been shown to drive an interferon-mediated cell death process in *Drosophila* [49], whose upregulation correlates with higher DENV replication [29]. Increased expression of both *SUMOE2* and *AeNopo* benefit DENV replication and therefore it bodes well with them being highly present in those families with higher infections. The opposite trend is seen for *smp-30* (Fig.3b) due to its involvement in oxidative stress protection, down regulated in highly infected individuals.

In addition to modulating host responses, DENV also must hijack host machinery to replicate efficiently [64, 70], but we did not find any difference in expression for genes involved in transcription such as the *Aedes mut-7* homolog or *rent1*. Both of these genes are involved in splicing complexes and RNA processing and control [71, 72].

### Adhesion and intracellular transport

In our study, we revealed the differential expression of a variety of adhesion molecules and intracellular transporters that DENV may utilize for entry and replication [73, 74]. *Rab5*, which has already been shown as required for flavivirus cell entry in humans [55], encodes a protein involved in vesicle formation and regulation of intracellular trafficking. We detected an increase in *Rab5* expression in families that harbor greater DENV loads, suggesting it may play a similar role for DENV entry in insects. Studies based on other vector-borne pathogens, including chikungunya and Venezuelan Equine Encephalitis viruses, have demonstrated the role of *Rab5* in promoting viral infection [56, 57].

The *aquaporin* gene is a member of a large family of transporters of water, with known roles in mitigating desiccation [75] and managing bloodmeal-induced diuresis [59]. The expression of *aquaporin* is commonly down regulated in a range of transcriptomic studies of host responses to DENV, Yellow Fever and West Nile viruses [12, 21]. Similar to transcriptomic studies, we find that it is lowly expressed in families with high DENV loads. However, the exact role of water transporters in blood-sucking insects and how they affect viral replication is still unknown. In *Drosophila*, *aquaporin* is primarily expressed in the carcass of the insect [76]. We hypothesize that if the expression pattern is similar in *Ae. aegypti*, downregulation of *aquaporin* may promote viral replication by altering the cellular water composition and osmosis of the body cavity.

### Metabolism

DENV uses host receptors and intracellular transporters to achieve infection, but it also relies on lipid rafts and modulation of the cell membrane composition to match that of the viral membrane and therefore facilitate viral entry to host cells [54, 77]. Our approach detected differential expression for genes involved in metabolism of lipids and sugars and possibly in the redistribution of such host resources. Among these metabolic genes, we detected the downregulation of α*-glucosidase* and *Pglym* in highly infected families. Studies suggest that α-glucosidase is proviral in humans [78, 79] and, as such, the downregulation of its expression is likely a host-induced anti-viral response. The downregulation detected for *Pglym* expression may not be due to its antiviral activity, but its position in the glycolysis pathway. Other genes involved on the breakdown of glucose have been reported to be key for viral control, such as aldolase [80]. However, in the same study, *Pglym* did not show antiviral properties. The modulation of metabolic genes may be caused by DENV-mediated redirection of resources inside the host [54]. Despite the importance of metabolic pathways to viral replication, other genes involved in metabolism were also found to be irrelevant for viral control, such as *sphingomyelin phosphodiesterase (SMase)*. *SMase* is a gene that specifically degrades sphingomyelin (SM), but also acts in response to cellular stresses through production of ceramide, which is linked to DENV infection responses [54]. This suggests that SMase may be acting early in infection, altering the cell outer membrane to produce a more curved membrane that favors DENV infection [81, 82]. However, we would not detect modulation of SMase given the late timepoint post infection we surveyed if SMase was not also directly affecting viral replication.

### Caveats

The design of our study presents some caveats that may limit its interpretation. The experimental conditions differ across the transcriptomic studies we surveyed and from the conditions in our study. Since some host responses are highly plastic, comparisons across different collection points may not be valid. Due to the destructive nature of the collections, the scale of breeding designs, and the sample size needed to achieve statistical power within and between families, only one time point (7dpi) could be assessed. Our results, while finding genes whose expression correlates with expression, do not speak to the excluded genes that could be relevant at different time points or in other tissues, etc. Additionally, we bypassed the midgut by injecting DENV-2 intrathoracically, which does not mimic a natural infection. Generally, the midgut is a physical host barrier that increases variability in susceptibility to the virus, as the ability to confront the infection will vary from mosquito to mosquito, even within the same viral-mosquito strain combination. For the purpose of our study, intrathoracic injections allowed us to focus solely in the variability of infection mediated by the carcass. This method also allowed control of the amount of virus delivered into each mosquito. Injection can cause changes in gene expression due to trauma itself, but any expression differences observed in our experiments cannot be due to trauma since all individuals received the same treatment. Regardless, these elicited responses tend to be short lived [12] and hence are likely to have returned to basal levels by sampling point.

## Conclusions

In conclusion, we found that natural genetic variation in vector competence within a single line of mosquitoes can be used to test for expression of gene candidates that vary with respect to viral control. This approach may offer a stepping stone from long lists of gene candidates produced in transcriptomic and other genome-wide expression studies prior to beginning more labor-intensive functional studies. It may also help to identify candidate genes not previously identified as antiviral. In this study, we have generated a list of 12 candidate genes that should be further examined as potential targets of gene modification to produce DENV-refractory mosquitoes (Table 2).

**Table 2:** Differentially expressed genes relevant for DENV control

Accession number	Gene name	Function	Direction of regulation
AAEL001022	*smp-30/regucalcin*	Senescence marker protein, Ca^2+^ binding domain	Down
AAEL001392	*defensin A-assoc*	Immunity (TOLL, Imd)	Down/Up
AAEL002413	*sphingomyelin*	Component of cellular membranes, reshuffling of which is important for virus intake	Down
AAEL003619	*–*	Putative Na/Cl transporter	Down
AAEL003787	*Nopo*	Zinc finger, ubiquitination	Up
AAEL004361	*alpha-glucosidase*	Molecule that mediates glycolysis, essential for virus replication	Down
AAEL007845	*Rab5*	Involved in endocytic trafficking of DENV	–
AAEL008108	*GB76c*	Transmembrane signalling molecule, GTPase activity	Down
AAEL009770	*SUMOE2*	Involved in sumoylation, process that stabilizes NS5 for replication	Up
AAEL011375	*trypsin*	Serine protease; effects of this kind of proteins are well described in the midgut	Down
AAEL011566	*–*	Putative adhesion molecule	Down
AAEL014108	aquaporin	Water transporter	Down

This summary table shows accession numbers, gene names, function and patterns of expression across families for those genes likely involved in DENV control

## Additional file

Additional file 1: Primer sequences used to test the expression of the different gene candidates. (PDF 47 kb)

## Data Availability

The datasets supporting the conclusions of the article are available in Figshare DOI 10.4225/03/5955dbeb34336

## Abbreviations

DENV: Dengue virus
Dpi: Days post infection
EIP: Extrinsic incubation period
