# Supplementary material for: Using genetic variation in Aedes aegypti to identify candidate anti-dengue virus genes
Source: BMC Infect Dis. 2019 Jul 4;19:580. doi: 10.1186/s12879-019-4212-z (PMC6611004; doi:10.1186/s12879-019-4212-z)
Supplement: Supplementary file 1 — Primer sequences used to test the expression of the different gene candidates. (PDF 47 kb) [file 12879_2019_4212_MOESM1_ESM.pdf]

**Table S1. –** Primer sequences used to test the expression of the different gene candidates.

| GENE ACCESSION | FW | RV | LENGTH PRODUCT (BP) | TM |
|---|---|---|---|---|
| **AAEL001022** | GCTACCGCTCATACGGACAC | CAGCACCATTTTCGTCATTG | 99 | 60 |
| **AAEL001156** | GAAGCAGTCCTCTGGAATCG | ATGTTGAAGGCGGAAATCAC | 160 | 60 |
| **AAEL001392** | ATGGTGGACATGCTGGAGTC | TCCGTGACCTCCTCCATAAG | 71 | 60 |
| **AAEL002413** | GCAGGTTTTTGGGGTGACTA | GGTGCTGGGTTCGTATCTGT | 85 | 60 |
| **AAEL002585** | ATCCCGTATCAGGAACAACG | CGTCGGCTACGAAATTGTCT | 115 | 60 |
| **AAEL003619** | AAGGTGGTCATCGGTATTGC | AGAAGGTGAACGCTTCCAGA | 148 | 60 |
| **AAEL003787** | GTAGCGAGCAATAGCGAACC | ACTCACTGGCCTGCAGTTCT | 76 | 60 |
| **AAEL004361** | GTCACGCTATGGGTTCATCC | CGTTCTCCTCGCTATTCCAC | 198 | 60 |
| **AAEL004861** | GAAAGCAGCGAAGAAACCAG | CAATGACACAGTCGGAATCG | 137 | 60 |
| **AAEL005064** | TCCTCTGGACACTCCAATCC | CTCGTCGATCTTGGTGTTGA | 112 | 60 |
| **AAEL005527** | GGAGCTGTTCGAGGAGTTTG | CTCGGCGATCGATATCTTGT | 76 | 60 |
| **AAEL006995** | GTCCTCTGTCCGAAATCCAA | TTCTACGACCATCACCAGCA | 92 | 60 |
| **AAEL007495** | GACATTTATCGCAGCCGAGT | AGCCATCTTGCCGTTATCTC | 94 | 60 |
| **AAEL007845** | ACACTGCTGGACAGGAAAGG | GTCGCTGAAGCTCTTTGACC | 138 | 60 |
| **AAEL008013** | ACGATGTCTCTATCCGCAAG | ATCCCTCTCCTTCCAGCACT | 76 | 60 |
| **AAEL008108** | TATGTCCGATGTCCCAAAGG | CAGGTGTCCCAGATGATGAG | 147 | 60 |
| **AAEL009317** | AACCGTCACCATCAGGAGAG | CGAACGGGACAGAAAAAGAG | 141 | 60 |
| **AAEL009602** | TTGGAGGATCGTTTCTGGAC | GTAGGCAGGGTATGCTGGAA | 98 | 60 |
| **AAEL009770** | TCAAGGACGACTACCCAACC | CCAGTCCTTCTCCTCATCCA | 119 | 60 |
| **AAEL011375** | GTCGGATTAGTGTCGGGATG | CGAAGATAGCCTGCGAGAAA | 136 | 60 |
| **AAEL011566** | GGACGAGAAGCTCAACGAAC | GGAGCAGATTCCACCACAAT | 147 | 60 |
| **AAEL011817** | GCTTGGCCCTGTTGTAATGT | TACGACCAACCGTTCAAACA | 76 | 60 |
| **AAEL012089** | GCCAATAAGAAGTCACCCTCTG | CGGTTTTGTCGTTTCCCTTT | 94 | 60 |
| **AAEL013712** | TGTGCAGCTTCTGGTCAAAC | GATGGCGGTTGACCTTCTTA | 100 | 60 |
| **AAEL014108** | CGCTACGTCCAGCTGTATTG | CCTCGATGAACGCTCCTAAG | 115 | 60 |
